# The Consumption of the Fibrous Fraction of *Solanum lycocarpum* St. Hil. Does Not Preserve the Intestinal Mucosa in TNBS-Induced Rats

**DOI:** 10.3390/foods13182949

**Published:** 2024-09-18

**Authors:** Amanda Maria Tomazini Munhoz Moya, Thaís Dolfini Alexandrino, Joseane Morari, Livia Mateus Reguengo, Licio Augusto Velloso, Raquel Franco Leal, Stanislau Bogusz Junior, Ana Paula Aparecida Pereira, Glaucia Maria Pastore, Juliano Lemos Bicas, Cinthia Baú Betim Cazarin

**Affiliations:** 1 School of Food Engineering, Universidade Estadual de Campinas, Rua Monteiro Lobato, 80, Campinas 13083-862, São Paulo, Brazil; amandamunhoz.moya@gmail.com (A.M.T.M.M.); tata_dolfini@hotmail.com (T.D.A.); liviareguengo@gmail.com (L.M.R.); pereira.anap23@gmail.com (A.P.A.P.); glaupast@unicamp.br (G.M.P.); bicas@unicamp.br (J.L.B.); 2 School of Medical Sciences, Universidade Estadual de Campinas, Rua Tessália Vieira de Camargo, 126, Campinas 13083-887, São Paulo, Brazil; morarij@gmail.com (J.M.); lavellos@unicamp.br (L.A.V.); rafranco@unicamp.br (R.F.L.); 3 São Carlos Institute of Chemistry (IQSC), University of São Paulo (USP), São Carlos 13566-590, São Paulo, Brazil; stanislau@iqsc.usp.br; 4 Faculty of Nutrition, Federal University of Mato Grosso, Avenida Fernando Correa da Costa, 2367, Boa Esperança, Cuiabá 78068-600, Mato Grosso, Brazil

**Keywords:** inflammatory bowel disease, ulcerative colitis, non-digestible carbohydrates, fruta-do-lobo

## Abstract

*Solanum lycocarpum* St. Hil. is considered a natural anti-inflammatory. In traditional medicine, it is used to reduce cholesterol levels in the treatment of obesity. Foods capable of conferring a protective and nutritious effect have been used to prevent or attenuate the clinical symptoms of inflammatory bowel diseases. Ulcerative colitis is a multifactorial inflammatory bowel disease. This study investigated the impact of the consumption of the fibrous fraction (FF) and resistant starch (RS) of fruta-do-lobo in an experimental model of colitis induced with the use 2,4,6-trinitrobenzene sulphonic acid (TNBS) in rats. The different colitis groups all experienced decreased weight gain, which could be linked to the inflammatory process (*p* = 0.603). Additionally, the experimental model led to increased oxidative stress, higher levels of pro-inflammatory cytokines, and the elevated gene expression of these cytokines. Despite this, consuming the fibrous fraction of fruta-do-lobo (RS and FF) did not appear to protect the animals against the inflammatory process. Regarding the expression of TNF-α, only the group treated with the drug mesalamine had a reduced serum level of this inflammatory marker (*p* = 0.03). Our results showed that the diet containing RS and FF did not protect the intestinal mucosa against TNBS inflammation. New studies on the variation in the time of consumption or the supplemented dose of fruta-do-lobo fibers could help to elucidate their effects in protecting the mucosa.

## 1. Introduction

In the period from 2012 to 2020, the incidence of ulcerative colitis in Brazil increased significantly from 5.7 per 100,000 to 6.9 per 100,000 (*p* < 0.0001) [1]. Ulcerative colitis (UC) is a specific inflammatory condition that affects the colon and rectum, causing inflammation and ulceration in continuous areas of the mucosa and submucosa [2]. While its exact cause is unknown, and a cure has not yet been found, scientific evidence suggests that genetic susceptibility, environmental factors, the immune system response, and the intestinal microbiota composition are linked to its development [3]. The disease is characterized by phases of remission and the recurrence of symptoms, ranging from mild colic, abdominal discomfort, and diarrhea to severe symptoms such as loss of appetite, weight loss leading to nutritional deficiencies, the presence of mucus in the stools, fever, severe rectal bleeding, and anemia [4,5]. 

The enteric homeostasis and control of the organism’s communication with the luminal environment, where intestinal microbiota is located, depends on maintaining the intestinal barrier’s selective permeability [6]. More precisely, changes in the tight junction protein expression occur in UC active periods, decreasing the barrier’s structural conformation, facilitating the translocation of pathogenic substances, and activating the innate immune system. Additionally, it generates an inflammatory response through the release of immunologic mediators by dendritic cells, lymph nodes, macrophages, and neutrophils, maintaining the state of a commensal relation with bacteria [7]. Thus, maintaining intestinal barrier integrity helps to avoid inflammatory disorders and benefits the normal intestinal transit of substances in the lumen [8].

The intestinal microbiota comprises hundreds of commensal bacteria, which interact with the host symbiotically, contributing to nutrition, metabolism, and the immune system. However, an imbalance in microbiota composition and diversity characterizes a dysbiosis condition, a serious issue that contributes to the development of the inflammatory status [9]. Additionally, inflammatory bowel diseases (IBDs) compromise patients’ quality of life due to their systemic complications and the adverse effects of pharmacological treatment [10]. 

Low dietary fiber intake is associated with increased non-communicable disease incidence, including IBDs [11,12]. The beneficial effects associated with the consumption of dietary fiber, including resistant starch, are related to the modulation of inflammation by decreasing the concentrations of inflammatory markers, improving intestinal transit, and excreting damaging substances [13,14]. 

Moreover, the chemical linkages present in dietary fiber’s structure and resistant starch limit the activity of the digestive enzymes, favoring their fermentation by the colon’s resident microbiota, especially bifidobacteria, which produces short-chain fatty acids (SCFAs) as a metabolite [15,16]. Among SCFAs, butyrate is used as an energy substrate by colonocytes and is a key mediator of intestinal anti-inflammatory effects, contributing to colon health [17]. Also, butyrate inhibits the activation of the nuclear factor kappa B (NF-κB), which has an important role in regulating the expression of the inducible nitric oxide (iNOS) enzyme pathway [18]. Furthermore, the literature has demonstrated that increased nitric oxide (NO) production is associated with oxidative stress and cell apoptosis in intestinal inflammation [19]. Thus, SCFAs can regulate the inflammatory process by modulating iNOS expression, thus becoming an important alternative for reducing NO levels in patients with inflammatory diseases [20].

*Solanum lycocarpum* St. Hill is a Brazilian fruit widely found in the Cerrado ecoregion. It is popularly known as “wolf’s fruit” or “fruta-do-lobo” as it represents up to about 50% of the maned wolf’s diet [21]. In folk medicine, it is used as a sedative to treat epilepsy, diabetes, and obesity, as well as to reduce cholesterol levels and renal and abdominal pain [22,23,24]. Regarding the proximate composition, its pulp flour has 23 g/100 g of fiber and 32 g/100 g of resistant starch, confirming that *fruta-do-lobo* has high levels of fiber [21,25]. Therefore, using foods able to modulate the microbiota and favor the intestinal anti-inflammatory status can be a strategy to prevent or mitigate the clinical symptoms of inflammatory bowel diseases, as well as other chronic diseases, such as cancer and diabetes [26,27]. Thus, this study aims to analyze the nutritional composition of the fibrous fraction and resistant starch of fruta-do-lobo and its impact on IBD induced by TNBS.

## 2. Materials and Methods

### 2.1. Fruta-Do-Lobo Resistant Starch and Fibrous Fraction Extraction

**Figure 1:**
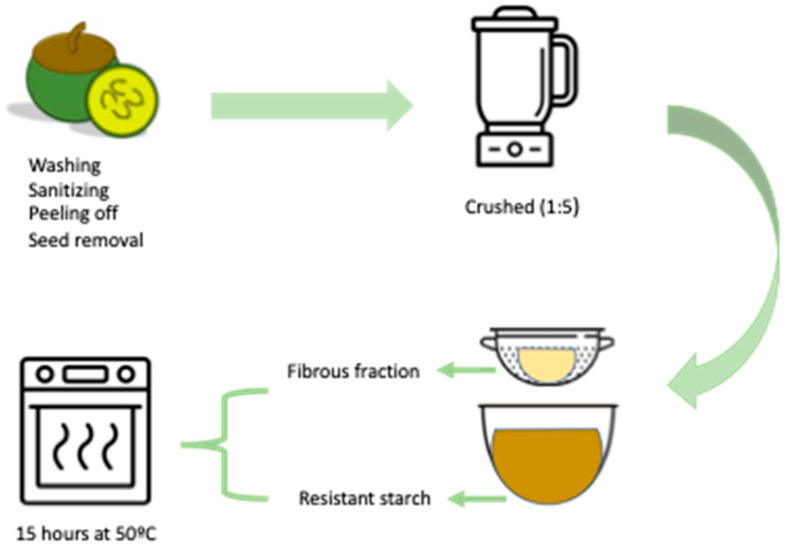
Schematic elucidating the process of obtaining resistant starch and the fibrous fraction of *fruta-do-lobo* (*Solanum lycocarpum* St. Hill). Adapted from Clerici et al. [21].

*Fruta-do-lobo* was collected in June 2017 in Carmo do Rio Claro (S20.555.209; W46.145.379), state of Minas Gerais, Brazil. Specimens were identified by Dr. Ingrid Koch and Dr. Leandro Giacomini, and a voucher specimen was deposited at the UNICAMP herbarium (UEC 197248). The access to Brazilian genetic heritage was registered at the Ministry of the Environment via SISGEN (protocol ADBCB71). The ripe fruits were manually washed with chlorinated water, followed by distilled and deionized water, cut into small pieces, and placed in a sodium bisulfite solution (200 mg/L). To remove excess bisulfite, they were washed, and the peel was carefully separated from the pulp using a sharp knife. The peel and pulp (seedless) were blended (model, brand) with distilled water (1:5 *v*/*v*) until a homogeneous suspension was obtained. To separate the flour portions, the suspension was sieved (mesh 22), retaining the fibrous fractions, and the liquid was kept under refrigeration overnight to decant the starch containing the resistant starch fraction. The starch was convectively dried at 50 °C for approximately 15 h in an oven (Ethik, 420-7D, Campinas, Brazil) (Figure 1).

The proximate composition of the fibrous fraction and resistant starch of the fruta-do-lobo determined by means of the quantification of moisture was determined by oven drying at 70 °C until a constant weight was reached; ash by the incineration of the organic material; and proteins (micro-Kjeldahl) based on official methods [28]. Lipids were determined with the method of Bligh and Dyer [29], total fibers by the enzymatic method [30], and total carbohydrates by the difference (Table 1). The available carbohydrate concentration was calculated according to the difference between 100 and the sum of the total percentage of proteins, lipids, ash, moisture, and fibers or resistant starch content, according to the following equation:

Total carbohydrate (%) = 100 − (protein + lipids + ash + moisture + fibers or resistant starch)

### 2.2. In Vivo Experimental Protocol

This study was conducted following the National Council for the Control of Animal Experimentation (CONCEA) and the Animal Research and Ethics Committee of the Universidade Estadual de Campinas (Brazil) (4941-1/2018) approval of the experimental protocol.

**Figure 2:**
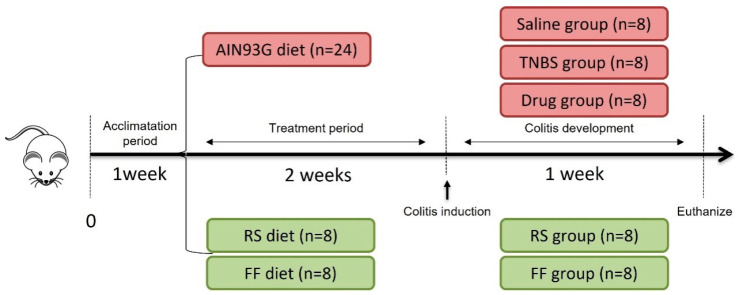
Schematic representation of the experimental protocol of interventions. Saline = normal diet (AIN-93G), healthy animals, and there was only the simulation of induction with saline solution; TNBS = colitis-induced negative control group; drug = control group with induced colitis that received the drug mesalazine; RS = group with induced colitis that received the resistant starch of fruta-do-lobo diet; FF = group with induced colitis that received the fibrous fraction of fruta-do-lobo diet.

Male Wistar rats (50 days old, weight ~309.9 ± 33.92 g) were housed under standard conditions of temperature (22 °C ± 2 °C), humidity (60–70%), and light/dark cycle (12/12 h). The rats were randomized into 5 groups (n = 8): saline (control, healthy), TNBS (control, TNBS), drug (control, mesalazine—100 mg/kg), Fruta-do-lobo resistant starch (RS), and Fruta-do-lobo fibrous fraction (FF). Animals were fed with a standard commercial diet (LABINA-PURINA) in the adaptation period. In the experimentation period, they were fed with an American Institute of Nutrition Growth diet (AIN-93G) [31], which could be supplemented or not with 3.8% RS (fruta-do-lobo resistant starch) or 3% FF (fruta-do-lobo fibrous fraction) ad libitum (Table 2 and Figure 2).

After two weeks of treatment, colitis was induced by the intracolonic administration of 2,4,6-trinitrobenzene sulphonic acid (TNBS) in the penultimate week of the experiment. The animals were anesthetized (ketamine hydrochloride 75 mg/kg and xylazine hydrochloride 10 mg/kg), and 10 mg of TNBS dissolved in 0.25 mL of 50% ethanol (*v*/*v*) was administered by intracolonic instillation using a Teflon cannula inserted 8 cm into the anus [32]. The same procedure was performed with the saline group by the intracolonic administration of 0.25 mL of 0.9% saline solution (*w*/*v*) instead of TNBS. After 7 days of induction, the animals were euthanized with anesthesia (ketamine chloride 300 mg/kg and xylazine chloride 30 mg/kg), and a cardiac puncture was performed on them. The blood samples were collected and centrifuged at 3500× *g* force for 15 min and stored at −80 °C until analysis. After exsanguination, the liver, spleen, and kidneys were removed, cleaned with saline solution, weighed, frozen in liquid nitrogen, and stored at −80 °C for future analysis.

### 2.3. Tissue Sampling and Analysis

The animals’ colons were removed, cleaned with 0.9% saline solution (*w*/*v*), measured and weighed, and then macroscopically evaluated for damage caused by inflammation on a scale of 0–10 [33]. Colon tissue was also microscopically analyzed; the samples were stored in 4% formalin, stained with hematoxylin and eosin, and evaluated on a scale from 0 to 50 [34]. 

Colon tissue homogenates were prepared in 75 mmol phosphate buffer (pH 7.4) using a Polytron homogenizer (MA102/Mini; Marconi, Piracicaba, SP, Brazil), and the supernatant was kept at −80 °C. The Bradford method was used to determine the protein concentration of colon homogenates [35]. 

Lipid peroxidation was performed by the thiobarbituric acid reactive substances (TBARS) assay using the colon homogenate [36]. Colon tissue samples were macerated in liquid nitrogen, and 10 mg/mL was sonicated in acetate buffer (pH 3.5) on ice. The samples were mixed with 8.1% sodium dodecyl sulfate (SDS, *w*/*v*) plus a working reagent (TBA, 20% acetic acid and 5% sodium hydroxide). After heating (95 °C for 60 min), they were maintained in an ice bath for 10 min and then centrifuged at 10,000× *g* for 10 min. The supernatant was read at 532 nm using a 96-well microplate. The results are expressed as nmol MDA equivalents/mg tissue.

The reduced glutathione (GSH) levels were determined in the phosphate buffer homogenates using Ellman’s reagent (5,5′-dithiobis-(2-nitrobenzoic acid)) [37]. GSH solution (2.5–500 nmol GSH/mL) was used as the standard curve, and absorbance was read at 412 nm. Reduced thiol contents are expressed in nmol GSH/mg protein. Also, the pro-inflammatory cytokines IL-1β and TNF-α were quantified in the tissue homogenate using a commercial ELISA kit (PeproTech, Ribeirão Preto, SP, Brazil).

Also, another portion of the colon was collected and stored in RNAlater^®^ (Sigma-Aldrich, St. Louis, MO, USA) for further analysis of the gene expression of the tight junction protein occlusion zone-1 (ZO-1, Rn.PT.58.37382645, Integrated DNA Technologies, Coralville, IA, USA) and tumor necrosis factor-alpha (TNF-α, Rn.PT.58.11142874, Integrated DNA Technologies) by quantitative polymerase chain reaction analysis in real time (RT-PCR) (Illumina, San Diego, CA, USA). Additionally, glyceraldehyde-3-phosphate dehydrogenase (GAPDH, PN4352338E, Thermo Fisher Scientific, Waltham, MA, USA) was used as a housekeeping gene.

### 2.4. Fecal Analyses

The content of SCFA was analyzed by gas chromatography using Agilent 6890N equipment coupled with a flame ionization detector (FID) and autosampler N10149 (Agilent, Santa Clara, CA, USA). A capillary column NukolTM (Supelco, Bellefonte, PA, USA) with 30 m × 0.25 mm i.d. × 0.25 µm was used to perform the SCFA separation and identification. Chromatographic conditions were injector and detector at 250 °C, split mode (1:10), 1 µL injection volume; helium carrier gas, 1 mL/min flow rate; initial temperature at 100 °C (0.5 min), increasing 8 °C/min until 180 °C (1 min), and then at an increasing rate of 20 °C/min until 200 °C for 5 min [38].

### 2.5. Statistical Analyses

ANOVA (analysis of variance) was used, followed by the Tukey’s significance test for parametric data, and the results are expressed as mean ± standard error. The Kruskal–Wallis test was used for non-parametric data, and the results are expressed as the median (range). All analyses considered an acceptable level of significance, *p* < 0.05.

## 3. Results and Discussion

Fiber-based foods are associated with health benefits. They contribute to achieving normal bowel function and prevent colonic diverticulosis and constipation [39,40]. Among them is resistant starch, a non-digestible carbohydrate fermented by the intestinal microbiota, which generates SCFA metabolites [41]. However, epidemiological studies show that fiber consumption in Brazil is low, as well as in other countries, which in many cases do not reach the recommendations of the Dietary References Intake (DRI) [42,43]. Therefore, improving fiber intake can be used as a complementary dietary strategy for the treatment of IBD.

**Figure 3:**
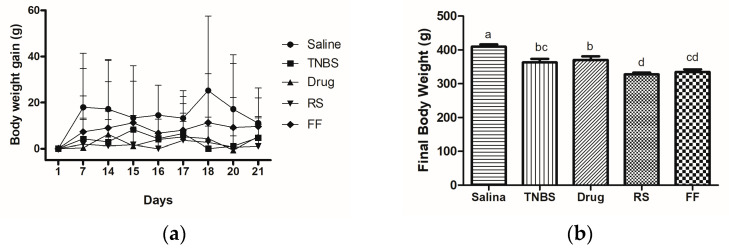
Effects of fruta-do-lobo resistant starch (RS) and fibrous fraction (FF) consumption on body weight gain (**a**) during the experimental protocol and the final weight (**b**) of the animals. The data are expressed as the mean SEM (n = 8 rats per group). Different superscript letters in the columns denote significant differences (*p* < 0.05) between the groups. Saline = control group; TNBS = colitis without treatment; drug = colitis treatment with mesalazine; RS = colitis treatment with fruta-do-lobo resistant starch; FF = colitis treatment with fruta-do-lobo fibrous fraction.

The induction of colitis probably interfered with the animals’ food intake since a decrease occurred in all groups except the saline group (Table 3). The saline group also showed the most significant weight gain during the experimental protocol, which was expected as it was the healthy control group. In contrast, in the other four groups, the animals’ weight was maintained or reduced (Table 3, Figure 3). This behavior is expected when associated with IBD, as appetite loss is one of the most frequent symptoms of ulcerative colitis, with consequent weight loss from malabsorption [44]. Moreover, the consumption of insoluble fiber is linked to reduced weight gain due to increased satiety, delayed gastric emptying, and decreased food intake [45,46]. In this sense, some in vivo experimental protocols have shown the effect of different sources of insoluble fiber in decreasing daily food intake and weight gain [47,48,49].

**Figure 4:**
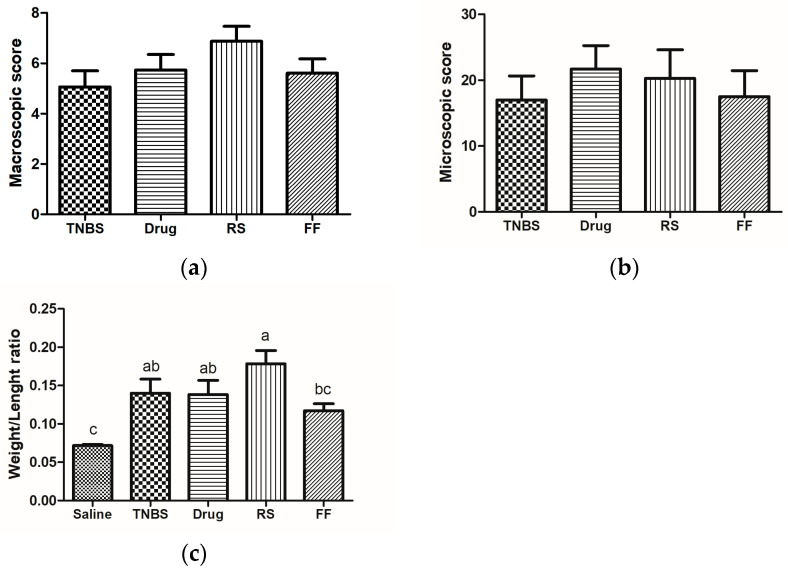
Effects of fruta-do-lobo resistant starch (RS) and fibrous fraction (FF) consumption on the intestinal mucosa macroscopic score (**a**), microscopic score (**b**), and weight/length ratio (**c**). The data are expressed as the mean SD (n = 8 rats per group). Different superscript letters in the columns denote significant differences (*p* < 0.05) among the groups. Saline = control group; TNBS = colitis without treatment; drug = colitis treatment with mesalazine; RS = colitis treatment with fruta-do-lobo resistant starch; FF = colitis treatment with fruta-do-lobo fibrous fraction.

**Figure 5:**
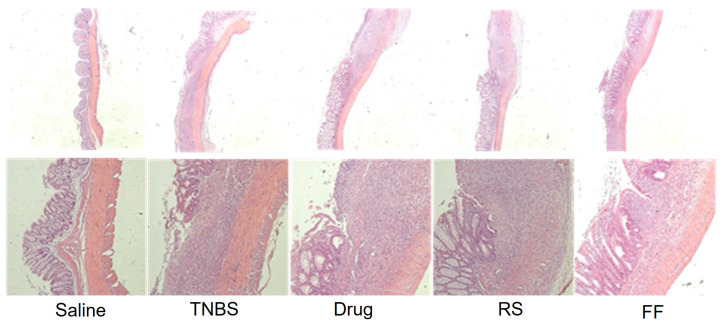
Effects of fruta-do-lobo resistant starch (RS) and fibrous fraction (FF) consumption in the histological analysis. Saline = control group; TNBS = colitis without treatment; drug = colitis treatment with mesalazine; RS = colitis treatment with fruta-do-lobo resistant starch; FF = colitis treatment with fruta-do-lobo fibrous fraction.

Colon tissue morphology is an important parameter in ulcerative colitis since it allows the identification of the injury degree, the presence of edema, and the thickening and shortening of the intestinal wall, which are some characteristics generated by inflammation in the intestinal mucosa and consequent of the incursion of pathogens [50]. Therefore, an increase in colon tissue weight is expected in the colitis-induced groups due to the presence of edema and inflammation, as well as tissue shortening. The weight/length ratio is widely used to evaluate these changes [51,52]. The evaluation of the weight/length ratio of the mucosa showed that consuming the FF and RS diet did not protect the animals’ intestinal mucosa from the thickening and shortening associated with the inflammatory process (Figure 4c). Additionally, no statistically significant difference was observed among the experimental groups in terms of macroscopic and microscopic damage to the intestinal mucosa (Figure 4a,b). As expected, the healthy saline group did not show morphological changes and thus was used as a control parameter for analyzing tissue morphology and structure (Figure 5).

**Figure 6:**
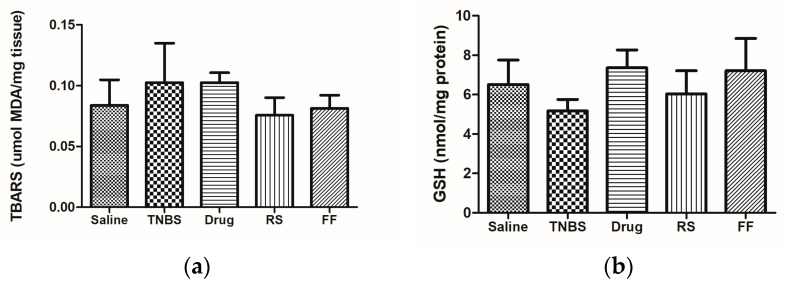
Effects of fruta-do-lobo resistant starch and fibrous fraction consumption on (**a**) TBARS and (**b**) reduced GSH content in the colonic tissue. The data are expressed as the mean SD (n = 8 rats per group). Saline = control group; TNBS = colitis without treatment; drug = colitis treatment with mesalazine; RS = colitis treatment with fruta-do-lobo resistant starch; FF = colitis treatment with fruta-do-lobo fibrous fraction.

Reactive oxygen metabolites play an important role in the pathogenesis of ulcerative colitis, as lipid peroxides and oxygen radicals damage intestinal mucosal cells. Therefore, TBARS is commonly used as an indicator of oxidative stress [53,54]. Studies show that the TBARS concentrations in untreated induced colitis rats’ colonic tissues were significantly higher than those in healthy groups, confirming oxidative stress in the experimental model [55,56]. The results did not show a statistical difference between the healthy and colitis groups. However, they suggest that there was more oxidative damage in the diseased animals, which is consistent with the findings in the literature (Figure 6a). The diets containing RS and FF did not effectively control oxidative stress in the animals’ colons. It is worth noting that TNBS is a very aggressive experimental model that causes severe damage to the epithelial tissue.

The intestinal mucosa has several antioxidant defense systems whose objective is to neutralize the deleterious effects of ROS’ continuous formation, in which GSH plays an important role in the cytoprotection of intestinal mucosa against oxidative stress [57,58]. 

We found no significant difference in the level of GSH in the colonic homogenates between the experimental groups (Figure 6b), which corroborates the TBARS data. 

The infiltration of colonic tissue by CD4 T cells and the secretion of potent pro-inflammatory cytokines such as TNF-α [59] is another consequence of the inflammation in IBDs. Many studies show increased serum levels of TNF-α in patients and animals with IBD [60,61,62,63]. 

**Figure 7:**
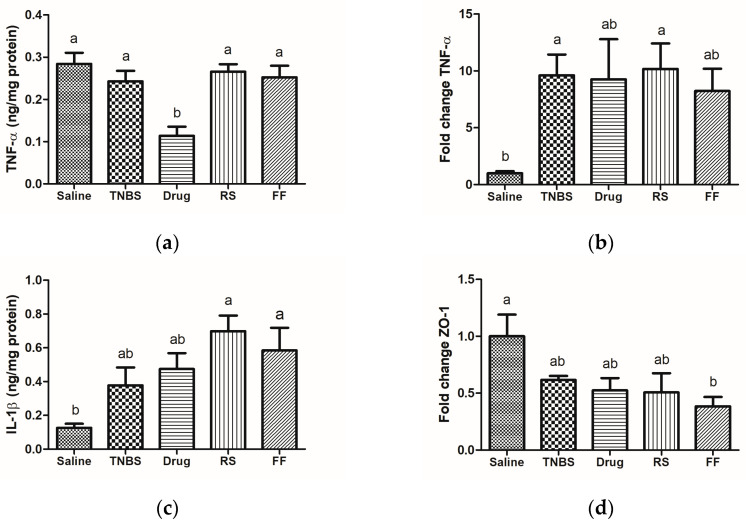
Effects of fruta-do-lobo resistant starch and fibrous fraction consumption on (**a**) TNF-α level, (**b**) TNF-α gene expression, (**c**) IL-1β level, and (**d**) zonula ocludens-1 gene expression. The data are expressed as the mean SEM (n = 8 rats per group). Different superscript letters in the columns denote significant differences (*p* < 0.05) between the groups. Saline = control group; TNBS = colitis without treatment; drug = colitis treatment with mesalazine; RS = colitis treatment with fruta-do-lobo resistant starch; FF = colitis treatment with fruta-do-lobo fibrous fraction.

The results of this study corroborate the scientific literature since all induced colitis groups presented higher expression and levels of pro-inflammatory cytokines in the intestinal mucosa. It is possible to observe that the mesalazine group (drug group) was the only group with reduced TNF-α levels (Figure 7a), confirming the effectiveness of the medication. Although elevated levels of TNF-α were observed in the ELISA assay, we observed that only the induced colitis groups showed an upregulated gene expression of this pro-inflammatory cytokine (Figure 7b). These data confirm the effectiveness of the experimental model in inducing inflammation in the intestinal mucosa of the animals. However, the consumption of RS and FF could not reduce the levels of pro-inflammatory cytokines (IL-1β and TNF-α) or their gene expressions, probably because the treatment could not inhibit the inflammatory pathway activation or counteract the damage induced in the mucosa.

Increased IL-1β levels in the intestinal mucosa are associated with the disease severity and the activation of oxidative-stress-responsive genes, which amplify and prolong inflammation. Therefore, finding increased IL-1β in patients or experimental models of IBD is expected [64]. A significant increase in IL-1β occurred in animals with colitis, and the consumption of FF and RS diets was not able to reduce these levels (Figure 7c). Although the present study did not find an association between FF and RS intake and the modulation of pro-inflammatory cytokines, some studies that used different types of fibers (prebiotic, insoluble fibers, and resistant starch from 8 to 42 days) showed a reduction in IL-1β both in animals and humans [65,66,67]. This disagreement may be explained by the type of fiber used, different doses, the treatment time, and the colitis induction method.

The intestinal barrier structure comprises tight junction proteins that control paracellular permeability. Tight junction proteins are also dynamically regulated by various extracellular factors and stimuli, apart from being associated with a susceptibility to develop intestinal diseases and with intestinal health [68]. Therefore, as a result of continuous changes that vary according to the type and amount of food consumed, drugs ingested, composition and load of luminal bacteria, and inflammatory status of the intestinal mucosa, we can observe changes in the expression and concentration of these proteins in luminal and serous compartments [8,69]. Moreover, the increased expression of pro-inflammatory cytokines, such as TNF-α and IL-1β, plays an important regulatory role in tight junction expression, thus increasing intestinal permeability and impairing barrier function. Therefore, the decrease in the expression of tight junctions in IBD may relate to the worsening of the inflammatory process [3]. Our results show that colitis induction promoted a decreased expression of the ZO-1 protein and that the consumption of the FF and RS diet could not prevent this change in the animals’ intestinal barrier (Figure 7d).

**Figure 8:**
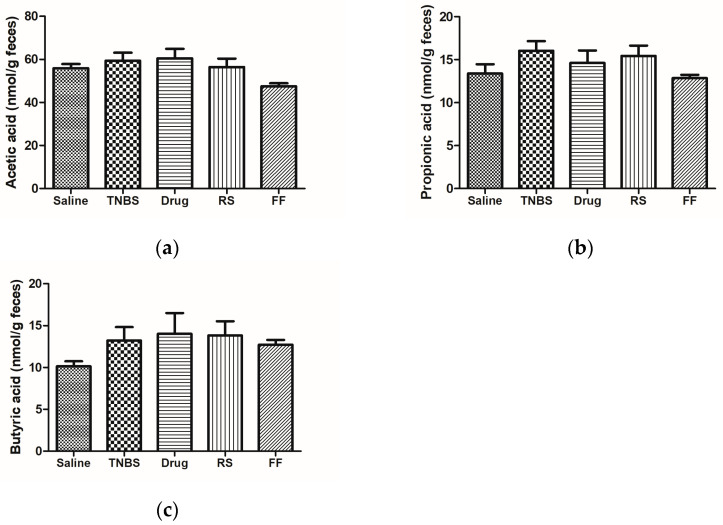
Effects of fruta-do-lobo resistant starch and fibrous fraction consumption on (**a**) acetic acid, (**b**) propionic acid, and (**c**) butyric acid production. The data are expressed as the mean SD (n = 8 rats per group). Saline = control group; TNBS = colitis without treatment; drug = colitis treatment with mesalazine; RS = colitis treatment with fruta-do-lobo resistant starch; FF = colitis treatment with *fruta do-lobo* fibrous fraction.

Dietary fibers, including resistant starch, can be used in the complementary treatment of IBDs since they are fermented in the large bowel, thus increasing the concentration of SCFAs, decreasing the colonic pH, and providing an unfavorable environment for the incursion and development of pathogens [65]. In this sense, about 95% of SCFAs that are products of microbiota fermentation are absorbed into the bloodstream, producing systemic immunomodulatory and anti-inflammatory properties, particularly useful for treating IBDs [70]. Furthermore, butyric acid protects the mucosa since enterocytes use it as a source of energy, thus contributing to maintaining intestinal and immunological homeostasis [71,72]. In vivo studies evaluating the effects of resistant starch and fiber in IBD models showed an increase in the production of SCFAs in groups that ingested these components compared to the groups without treatment [58,65,73,74]. The consumption of RS and FF did not change the production of SFCAs in the experimental groups; the RS group showed higher production rates than the FF group, probably because of the fiber characteristics and chemical structures (Figure 8). The FF has a lower capacity to produce SCFA because of its water insolubility characteristic, unlike resistant starch. On the other hand, resistant starch has properties like those of soluble fibers and thus is fermented and capable of producing more SFCAs during fermentation. As the RS fraction used in diet supplementation does not correspond 100% to resistant starch (Table 1), we believe that possibly increasing the supplemented dose in the animals’ diet could have been more effective in increasing SCFA production.

## 4. Conclusions

This study showed that the TNBS experimental model can cause injury and inflammation in the intestinal mucosa of animals. However, the consumption of fibers (RS and FF) added to the diet did not reduce the inflammation-induced damage. Therefore, further research on adjusting the consumption time or increasing the supplemented dose could be a potential approach to assess the impact of fruta-do-lobo fiber consumption on the intestinal mucosa, microbiota modulation, formation of metabolites, and mucosal permeability. This could also involve evaluating other proteins responsible for maintaining the intestinal barrier.

## Figures and Tables

**Table 1:** Proximate composition of resistant starch (RS) and fibrous fraction (FF) content of fruta-do-lobo (*Solanum lycocarpum* St. Hill).

Components	RS	FF
	(g/100 g)	
Moisture	8.66 ± 0.07	0.35 ± 0.07
Lipids	0.24 ± 0.00	0.18 ± 0.02
Ash	0.12 ± 0.02	1.69 ± 0.10
Protein	0.68 ± 0.02	3.76 ± 0.06
Total carbohydrates	60.51	46.08
Total fibers	-	47.94 ± 0.27
Insoluble fibers	-	47.38 ± 0.01
Soluble fibers	*	0.56 ± 0.01
Resistant starch	29.79 ± 0.61	-

Data are expressed as means ± SD. The assays were performed in triplicate. * Some soluble dietary fibers may have solubilized during starch extraction, but it was not possible to perform such quantification.

**Table 2:** Formulation and proximate composition of experimental diets.

	Control	RS	FF
Corn starch (g)	457.8	429.0	429.0
Casein (12% protein) (g)	139.7	150.0	150.0
Dextrinized corn starch (g)	132.0	142.5	142.5
Sucrose (g)	100.0	108.0	108.0
Soy oil (g)	70.0	70.0	70.0
Cellulose (g)	50.0	50.0	50.0
Fruta-do-lobo resistant starch (3.8%) (g)	-	38.0	-
Fruta-do-lobo fibrous fraction (3%) (g)	-	-	30.0
Mineral mix (g)	35.0	35.0	35.0
Vitamin mix (g)	10.0	10.0	10.0
L-cystine (g)	3.0	3.0	3.0
Cholinebitartrate (g)	2.5	2.5	2.5
Butylhydroquinone (g)	0.014	0.014	0.014
Total (g)	1000	1038	1030
Proximate Composition			
Moisture (%)	6.2 ± 0.15 ^c^	6.7 ± 0.11 ^b^	7.5 ± 0.08 ^a^
Ash (%)	2.4 ± 0.07	2.2 ± 0.10	2.3 ± 0.24
Lipids (%)	6.1 ± 0.57	6.4 ± 0.08	6.3 ± 0.27
Protein (%)	12.5 ± 0.39 ^a^	11.4 ± 0.47 ^b^	11.3 ± 0.29 ^b^
Total carbohydrate (%)	72.8	73.3	72.6
Calories (kcal) *	396.1	396.4	392.3

Fruta-do-lobo resistant starch diet (RS) and fruta-do-lobo fibrous fraction diet (FF). Data are expressed as mean ± SEM. Different letters represent the statistical difference (ANOVA followed by Tukey’s test, *p* < 0.05). Diet formulation adapted from Reeves et al. [31] * Calorie content of the diet was estimated by protein and carbohydrate content multiplied by 4 and lipids by 9.

**Table 3:** Effects of fruta-do-lobo (*Solanum lycocarpum* St. Hill) resistant starch (LRS) and fibrous fraction (LRE) on food intake and body and tissue weight.

	Saline	TNBS	Drug	RS	FF
Liver (g)	16.6 ± 1.8	14.5 ± 1.93	15.6 ± 2.30	15.0 ± 1.27	14.3 ± 1.08
Spleen (g)	1.2 ± 0.28	1.2 ± 0.20	1.2 ± 0.20	1.5 ± 0.40	1.1 ± 0.14
Cecum (g)	2.9 ± 0.59	3.6 ± 0.36	3.3 ± 0.50	2.6 ± 0.33	4.1 ± 0.46
Body weight gain (g)	129.9 ± 34.72 ^a^	72.0 ± 18.19 ^b^	71.7 ± 25.32 ^b^	78.0 ± 7.92 ^b^	81.7 ± 14.49 ^b^
Food intake (mg/kg bw)	25.4 ± 3.4	21.0 ± 5.7	21.3 ± 5.9	17.9 ± 6.4	21.3 ± 3.6
Energy intake_cal_	104.1 ± 14.0	86.0 ± 23.37	87.3 ± 24.1	73.5 ± 26.1	87.5 ± 26.1

Saline = AIN-93G diet; TNBS = AIN-93G diet; drug = AIN-93G + mesalazine; RS = AIN-93G diet + 3.8% fruta-do-lobo resistant starch; FF = AIN-93G diet + 3% fruta-do-lobo fibrous fraction. The data are expressed as the mean ± SEM (n = 8 rats per group). Different superscript letters in the line denote significant differences (*p* < 0.05) between the groups.

## Data Availability

The original contributions presented in this study are included in this article; further inquiries can be directed to the corresponding author.
